# The Addition of Zinc to the ICIE16-Bioactive Glass Composition Enhances Osteogenic Differentiation and Matrix Formation of Human Bone Marrow-Derived Mesenchymal Stromal Cells

**DOI:** 10.3390/biomimetics9010053

**Published:** 2024-01-18

**Authors:** Felix Rehder, Marcela Arango-Ospina, Simon Decker, Merve Saur, Elke Kunisch, Arash Moghaddam, Tobias Renkawitz, Aldo R. Boccaccini, Fabian Westhauser

**Affiliations:** 1 Department of Orthopaedics, Heidelberg University Hospital, Schlierbacher Landstraße 200a, 69118 Heidelberg, Germanymerve.saur@med.uni-heidelberg.de (M.S.);; 2 Institute of Biomaterials, University of Erlangen-Nuremberg, Cauerstr. 6, 91058 Erlangen, Germany; 3 PrivatÄrztliches Zentrum Aschaffenburg, Frohsinnstraße 12, 63739 Aschaffenburg, Germany

**Keywords:** ICIE16-bioactive glass, zinc, bone tissue engineering, human mesenchymal stromal cells, osteogenic differentiation

## Abstract

An ICIE16-bioactive glass (BG) composition (in mol%: 49.5 SiO_2_, 6.6 Na_2_O, 36.3 CaO, 1.1 P_2_O_5_, and 6.6 K_2_O) has demonstrated excellent in vitro cytocompatibility when cultured with human bone marrow-derived mesenchymal stromal cells (BMSCs). However, its impact on the development of an osseous extracellular matrix (ECM) is limited. Since zinc (Zn) is known to enhance ECM formation and maturation, two ICIE16-BG-based Zn-supplemented BG compositions, namely 1.5 Zn-BG and 3Zn-BG (in mol%: 49.5 SiO_2_, 6.6 Na_2_O, 34.8/33.3 CaO, 1.1 P_2_O_5_, 6.6 K_2_O, and 1.5/3.0 ZnO) were developed, and their influence on BMSC viability, osteogenic differentiation, and ECM formation was assessed. Compared to ICIE16-BG, the Zn-doped BGs showed improved cytocompatibility and significantly enhanced osteogenic differentiation. The expression level of the osteopontin gene was significantly higher in the presence of Zn-doped BGs. A larger increase in collagen production was observed when the BMSCs were exposed to the Zn-doped BGs compared to that of the ICIE16-BG. The calcification of the ECM was increased by all the BG compositions; however, calcification was significantly enhanced by the Zn-doped BGs in the early stages of cultivation. Zn constitutes an attractive addition to ICIE16-BG, since it improves its ability to build and calcify an ECM. Future studies should assess whether these positive properties remain in an in vivo environment.

## 1. Introduction

When Larry Hench and co-workers reported, for the first time, the 45S5-bioactive glass (BG) composition (in mol%: 46.1 SiO_2_, 24.4 Na_2_O, 26.9 CaO, and 2.6 P_2_O_5_) in 1969, they introduced a new era in the field of bioactive materials, providing strong bonding to the bone tissue [1]. Not only does the material itself affect its surrounding tissue, but the ionic dissolution products (IDPs) released from the BG have an impact on the signaling pathways in the broader environment, facilitating the processes of cells, such as osteogenic differentiation [2,3,4,5]. In recent decades, several new BG compositions have been developed in order to extend their fields of applications and to further improve their biological properties [6]. For example, when Elgayar et al. introduced the ICIE16-BG composition (in mol%: 49.5 SiO_2_, 6.6 Na_2_O, 36.3 CaO, 1.1 P_2_O_5_, and 6.6 K_2_O) in 2004 [7], they described an extended sintering window, leading towards less crystallization in comparison to that of the 45S5-BG composition, making the ICIE16-BG composition attractive for producing scaffolds, while maintaining the amorphous characteristics of the BG [8,9,10,11]. Furthermore, compared to the other BG compositions, the network connectivities of ICIE16-BG and 45S5-BG are comparable, allowing the release of IDPs into the BGs’ surrounding environment and the rapid development of a hydroxyapatite layer on the BG surface [8]. The release of IDPs makes BGs versatile vectors to deliver ions with a certain therapeutic activity to the designated field of application [5]. ICIE16-BG, therefore, constitutes an attractive candidate to be substituted with ions of certain biological activity in order to further adapt and improve its biological properties, for example, in bone tissue engineering (BTE) applications [12]. When compared directly to the 45S5-BG composition, ICIE16-BG showed improved cytocompatibility and comparable osteogenic properties, but its impact on the formation and maturation of a primitive osseous extracellular matrix (ECM) was limited in vitro [13]. The osteogenic properties of ICIE16-BG, in particular, its influence on ECM formation and maturation, might be enhanced by adding therapeutically active ions that specifically facilitate these biological properties.

One attractive candidate ion to be added to ICIE16-BG is zinc (Zn), since it plays an important role in various metabolic pathways, including, but not limited to, homeostasis, immune functions, and aging [14,15]. It furthermore acts as a key factor in bone mineralization and growth, for example, by stimulating osteoblast protein synthesis, while downregulating osteoclastic bone resorption [16]. Zn also supports osseous extracellular matrix (ECM) modeling and keeps the bone metabolism in balance [17]. Zn has already proven its advantageous qualities as a part of the biomaterials intended for BTE applications in several investigations before [18,19,20]. However, the impact of Zn incorporated into the ICIE16-BG composition on human bone marrow-derived mesenchymal stromal cells (BMSCs) has not been examined yet. Hence, in this study, 1.5 mol% or 3 mol% ZnO was added to the ICIE16-BG composition in exchange for CaO, resulting in two new BG compositions, namely 1.5 Zn-BG and 3 Zn-BG (in mol%: 49.5 SiO_2_, 6.6 Na_2_O, 34.8/33.3 CaO, 1.1 P_2_O_5_, 6.6 K_2_O, and 1.5/3.0 ZnO). The influence of the IDPs of both the Zn-substituted BG compositions on BMSCs was compared to that of the IDPs of the unmodified ICIE16-BG. This study focuses on the evaluation the BGs’ impact on the formation and maturation of a primitive osseous ECM. Furthermore, the impact on cell viability and osteogenic differentiation at the cellular and genetic levels was assessed.

## 2. Material and Methods

### 2.1. BG Production and Characterization

The melt-quench route was used to produce BGs from analytical grade reagents, such as CaCO_3_ (Honeywell Fluka, Dreieich, Germany), NaCO_3_ (Honeywell Fluka), K_2_CO_3_ (Alfa Aesar, Dreieich, Germany), CaHPO_4_·2H_2_O (Acros Organics, Geel, Belgium), ZnO (Sigma-Aldrich, Steinheim, Germany), and commercial-grade Belgian quartz sand (SiO_2_). The glasses were melted in Pt crucibles for 1.5 h at 1420 °C before being casted in graphite molds and annealed for 1 h at 520 °C. The melting process was then repeated to ensure homogeneity. The glasses were crushed and milled to produce fine particles using a Jaw Crusher (Retsch, Haan, Germany) and a planetary ball mill (Retsch), respectively. Furthermore, the BGs underwent a heat treatment process for glass sintering for 1.5 h at 690 °C at a heating rate of 2 °C/min. This process aimed to replicate the heat treatment during the production of 3D scaffolds. The sintered materials were ground into fine powder and sieved to 100 microns. The compositions of experimental glasses are shown in Table 1. The morphology of the glass particles was examined with a scanning electron microscope (SEM, Auriga, Carl-Zeiss, Jena, Germany) using a voltage of 1.5 kV. The approximate particle size was obtained by measuring around 200 particles from SEM pictures using ImageJ (U.S. National Institutes of Health, Bethesda, MD, USA).

### 2.2. Ion Release from BGs

The dissolution of the glasses was carried out over a period of 21 days in cell culture medium (CCM). Glass particles were immersed in Dulbecco’s modified Eagle’s medium (DMEM, Thermo Fisher, Dreieich, Germany) in a concentration of 1.5 mg/mL and placed in an orbital shaker at 90 rpm and 37 °C. The ionic concentrations of silicon and Zn in the supernatants were analyzed with an inductively coupled plasma-optical emission spectrometer (ICP-OES, Optima 8300, Perkin Elmer, Hamburg, Germany).

### 2.3. Cell Harvesting and Ethical Approval

The BMSCs were harvested from a 20-year-old male donor that underwent surgery at Heidelberg University Hospital. The harvesting and use of the cells for the purposes of this study was approved by the responsible ethics board of the Medical Faculty of the University of Heidelberg (approval number: S-340/2018).

### 2.4. BMSC Isolation and Cultivation

The BMSCs were harvested from bone marrow and subsequently isolated following well-established protocols [13]. In short, bone marrow was obtained during surgery using a heparinized syringe. The heparinized bone marrow components were subsequently layered onto Ficoll-Paque Plus (GE Healthcare Europe, Freiburg, Germany) for phase separation. After centrifugation and repeated washing, the mononuclear cells were transferred to T75 culture flasks (Nunc, Roskilde, Denmark), which were coated with 0.1% gelatin (Sigma-Aldrich). Cultivation occurred under standard cell culture conditions (37 °C and 5% CO_2_ in a humidified atmosphere) in expansion medium (EM) that consisted of high-glucose DMEM supplemented with 12.5% fetal calf serum (FCS), 2 mM L-glutamine, 1% non-essential amino acids (NEAA), 50 μM β-mercaptoethanol (all Thermo Fisher), 100 μg/mL penicillin/streptomycin (PenStrep; Biochrom, Berlin, Germany), and 4 ng/mL fibroblast growth factor 2 (Abcam, Berlin, Germany). The first exchange of medium was performed after 24 h of cultivation to remove the non-adherent cells. The EM was changed every third day until 80% confluency was observed, and the cells were thereupon passaged. Experiments were conducted with cells in passages 4 and 5.

### 2.5. Cell Cultivation and Conditioning of CCM with BGs

**Figure 1:**
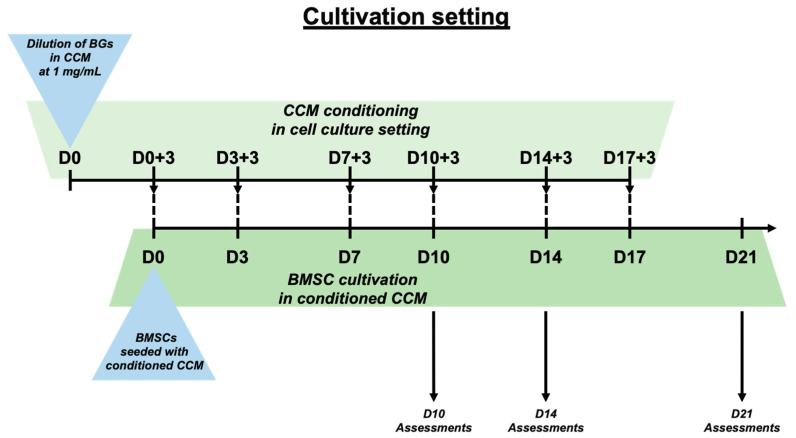
Schematic presentation of cultivation setting and the generation of BG-conditioned media. CCM changes were conducted on D3, D7, D10, D14, and D17.

The BMSCs were grown in an indirect cultivation under exposure to the BGs’ IDPs in the form of a BG-conditioned cell culture medium (CCM). The conditioning of the medium was performed by adding 1 mg of BG per 1 mL of CCM. The CCM consisted of high-glucose DMEM supplemented with 10% FCS and 100 µg/mL PenStrep (all Thermo Fisher). After 72 h, the CCM, including the ionic dissolution products, was collected, centrifuged, filtered with 0.22 µm syringe filters (Sigma-Aldrich), and then used for seeding the BMSCs (Figure 1). The BMSCs were seeded with the conditioned medium in a density of 18,400 cells/cm^2^. The BMSCs in a control group were cultivated in the same manner using a regular (non-conditioned) CCM. The medium was changed twice a week using regular CCM in the control group or conditioned CCM in the intervention groups as described above. Measurements were conducted after 10 (D10), 14 (D14), and 21 (D21) days.

### 2.6. Combined ALP Activity and Cell Viability Assay

Cell viability is assessed by quantifying the hydrolysis of fluorescein diacetate (FDA; Sigma-Aldrich) to the green fluorescent fluorescein that correlates with cell metabolism, as well as with the cell number. 4-methylumbelliferyl phosphate (4-MUP), on the other hand, is converted by the alkaline phosphatase (ALP) into blue fluorescing 4-methylumbelliferone (4-MU), and therefore, correlates with the enzyme’s activity, which serves as indicator of cellular osteogenic differentiation, since BMSCs increase ALP production when developing towards osteoblasts. Briefly, the CCM was removed, and the cells underwent washing with DPBS. Afterwards, the sample was stained with FDA substrate solution (0.1 mg/mL FDA in acetone (Carl Roth, Karlsruhe, Germany) 1:50 diluted in PBS) for 5 min at 37 °C. Following another washing procedure with DPBS, the samples were lyzed by using 0.5% Triton-X-100 (Sigma-Aldrich) in aqua dest. for 5 min at standard cell culture conditions. Thereafter, a solution of 4-MUP substrate (100 µM 4-MUP (Thermo Fisher) in ALP assay buffer (75 mM TRIS pH 9.3, 1.5 mM MgCl_2_, and 0.15 mM ZnCl_2_ (all Carl Roth)) was added to the aliquots of lysates, and the lysates were incubated for another 15 min at 37 °C. After incubation, FDA fluorescence was quantified using a fluorescence microplate reader (Wallac 1420 Victor 2; Perkin Elmer, Waltham, MA, USA), measured at 485/530 nm (ex/em). 4-MUP fluorescence was measured at 360/440 nm (ex/em). A standard dilution series ranging from 10 mU down to 0.16 mU ALP was plotted using shrimp alkaline phosphatase (Thermo Fisher) as the ALP standard working solution (0.4 mU/µL). For normalization purposes, the ALP activity of each sample was divided by the FDA fluorescence intensity of the respective samples.

### 2.7. Cell Growth Patterns and Qualitative Evaluation of Cell Viability

To observe cell morphology and viability, the cells were stained using FDA for the detection of viable cells and propidium iodide (PI; Thermo Fisher) for the visualization of compromised cells. FDA freely enters the cell and is enzymatically converted into the green fluorescent fluorescein, while PI is not able to pass the unimpaired cell membranes, and hence, only intercalates with the DNA of non-viable cells. The CCM was removed, and the staining solution (8 µg/mL FDA and 20 µg/mL PI in DPBS) was added for 5 min at 37 °C. Imaging was conducted with an Olympus IX-81 inverted fluorescence microscope (Olympus, Hamburg, Germany). Finally, the green (FDA) and red (PI) photographs were assembled by using Image J (U.S. National Institutes of Health).

### 2.8. Gene Expression Analysis via qPCR

By running a quantitative real-time polymerase chain reaction (qPCR), the expression levels of genes that encode for relevant proteins of the osseous ECM, namely Osteopontin (OPN), Osteocalcin (OCN), and Osteonectin (ON), were examined. Tyrosine 3-Monooxygenase/Tryptophan 5-Monooxygenase Activation Protein Zeta (YWHAZ) served as the endogenous control gene. Guided by the manufacturer’s instructions, total RNA was isolated using the PureLink RNA Mini Kit (Thermo Fisher). The reverse transcription from RNA into cDNA was performed using a High-Capacity RNA-to-cDNA Kit (Thermo Fisher). Finally, qPCR was performed using the primers specified in Table 2 in combination with PowerUp SYBR Green Master Mix (Thermo Fisher). The quantification of gene expression was calculated with the ΔΔCT method by referring the ΔCT values of the treated trials to the control.

### 2.9. Determination of Collagen Accumulation via Sirius Red Staining

Collagen production was quantified using Sirius Red, which specifically binds to collagen. The cells were fixed by adding 4% paraformaldehyde (PFA, Merck, Darmstadt, Germany) in PBS for 1 h at room temperature. Before adding the Sirius Red solution (1 mg/mL Sirius Red F3BA (Chroma-Gesellschaft, Münster, Germany) diluted in 1.3% picric acid (Sigma Diagnostics Inc., Livonia, MI, USA) in aqua dest.), PFA was removed, which involved three washing procedures with aqua dest. After 1 h of staining at room temperature, 0.01 M HCl (Carl Roth) was added to the samples and carefully removed. This washing step was repeated three times to remove the unbound staining solution. Subsequently, bound Sirius Red was eluted by adding 0.1 M NaOH (Carl Roth) for 30 min, while placed on a plate shaker. Optical density was measured with a microplate reader (Autobio PHOmo; Autobio Diagnostics, Zhengzhou, China) at 492 nm. For the quantitative determination of bound Sirius Red, a standard dilution series ranging from 90 µg down to 0 µg Sirius Red solution was plotted.

### 2.10. Determination of ECM Calcification via Alizarin Red S Staining

The extend of ECM calcification correlates with the grade of ECM maturation. The cells were fixed with 70% ethanol (Serva Electrophoresis, Heidelberg, Germany) in aqua dest. for 20 min at 6 °C after the CCM was discarded, and the cells were rinsed with DPBS beforehand. The fixation solution was removed, and the cells were once more washed with aqua dest. before 0.5% Alizarin Red S (Waldeck GmbH & Co. KG, Muenster, Germany) in aqua dest. was added for 10 min at room temperature. The supernatant of the staining solution was removed, and the cells underwent another washing procedure with aqua dest. to ensure only the bound stains remained. For quantification, the colored calcium was dissolved by adding 10% cetylpyridiniumchloride (Sigma-Aldrich) solution containing 10 mM sodiumdihydrogenphosphate (AppliChem, Darmstadt, Germany). Optical density was measured with a microplate reader (Autobio PHOmo) at 570 nm. For the quantitative determination of bound Alizarin Red S, a standard dilution series ranging from 90 µg down to 0 µg Alizarin Red S solution was plotted.

### 2.11. Statistics

Statistical analyses were performed using IBM SPSS Statistics (Version 25; IBM, Armonk, NY, USA). The results were statistically evaluated by performing a Kruskal–Wallis test and the Mann–Whitney-U test subsequently. *p*-values of <0.05 were regarded as significant. Due to its exploratory design, no correction for multiple testing was conducted. Graphs were created using Microsoft Excel and Microsoft PowerPoint (both Microsoft, Redmond, WA, USA). The values are shown as rounded means with standard deviation where suitable. The experiments were designed using *n* = 5 replicates for each assay. All quantitative measurements were completed in technical duplicates.

## 3. Results

### 3.1. Morphological Characterization of Synthesized BGs

**Figure 2:**
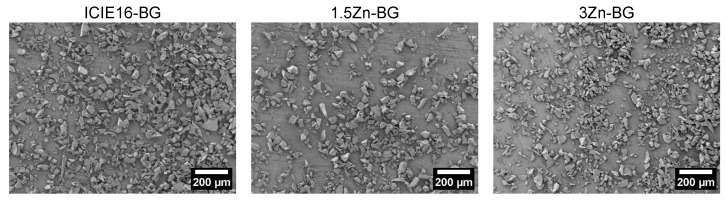
SEM pictures of BG particles.

The BGs particles exhibited a distinct polyhedral-like shape [21,22], which is typical of the granules produced using the melt-quenching method. As shown in Figure 2, there was no difference in terms of morphology and size between the tested compositions, resulting in average particle size values of 37 ± 14, 34 ± 13 and 35 ± 13 µm for the ICIE16-BG, 1.5ZnBG, and 3Zn-BG, respectively.

### 3.2. Ion Release Profiles of the BGs

**Figure 3:**
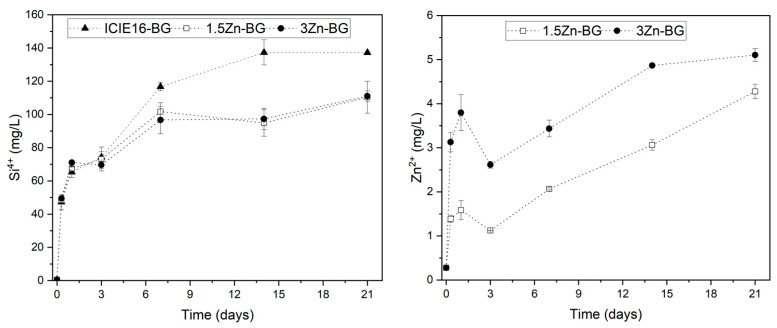
Release profiles of silicon and zinc ions in DMEM.

Ion release profiles from the BGs are shown in Figure 3. Until the third day of incubation, the degrees of silicon release were equivalent across all the compositions. Subsequently, a significant difference was observed between the ICIE16-BG and the Zn-doped variations, with a larger release of Si^4+^ from the ICIE16-BG over the remaining incubation time, implying a faster dissolution rate of the glass network. Furthermore, no difference was seen between the 1.5Zn-BG and 3Zn-BG. This effect has been reported for Zn-containing silicate glasses and may possibly be explained by the complexation of the glass network driven by the tetrahedral coordination that Zn species adopt, leading to the copolymerization of the network with SiO_4_ tetrahedra [23,24]. Additionally, the intermediate role of zinc in the glass network likely contributes to a more stable glass with the presence of stronger bonds such as Zn-Si compared to Ca-Si. This behavior has been reported with a dependent ZnO concentration, in which large amounts of ZnO lead to glasses with a higher resistance to dissolution [24,25]. In relation to the release of Zn, it was anticipated that the 3Zn-BG would exhibit a greater concentration of release compared to the 1.5Zn-BG, resulting in the doubling of concentrations throughout all the specified time intervals. The release of Zn exhibited a gradual increase over a period of 21 days, indicating that the release of this ion could occur even over extended incubation periods.

### 3.3. BMSCs’ Viability under Treatment with BGs

**Figure 4:**
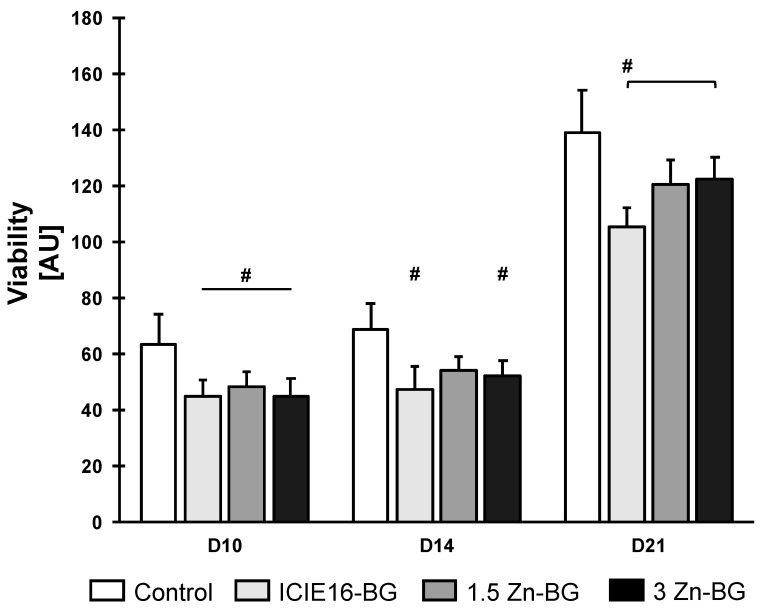
BMSC viability. Values show fluorescence intensity (means with standard deviation) and are expressed in arbitrary units (AU). Values labeled with # are significantly different compared to the control group. In case of multiple significant differences compared to the control group, the respective groups are labeled with a line and #. Brackets highlight significant differences between the two groups encompassed by the brackets.

The viability of BMSCs increased slightly from D10 to D14 and showed a considerable increase until D21 (Figure 4). The control group showed the most viability across the observation period. No significant difference was measured among the BG-groups on D10 and D14. On D21, however, the cells treated with the 3Zn-BG showed significantly more viability than those treated with the ICIE16-BG.

### 3.4. Growth Patterns of BMSCs Influenced by the BGs

**Figure 5:**
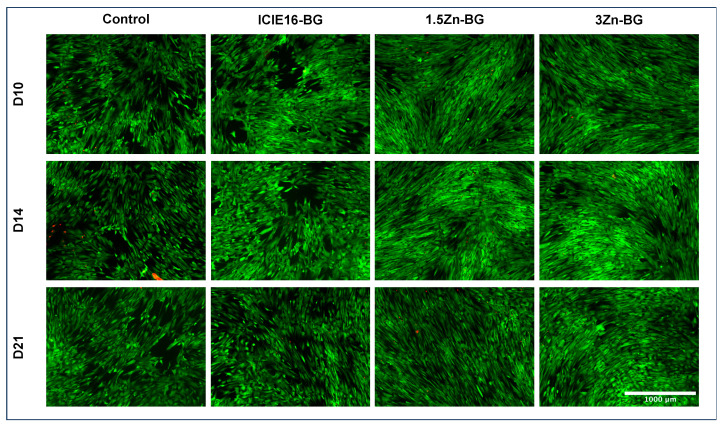
Fluorescence microscopy-imaging showing cell growth patterns. Coloring was performed via Image J. Scale bar was set to 1000 µm and is applicable to all images. The green fluorescent FDA stains viable cells, while the red fluorescent PI stains comprised cells.

Both the Zn-doped BGs showed a denser cell layer on D10 compared to the control and ICIE16-BG groups (Figure 5). From D14 on, the layers of BMSCs were confluent, independent from the treatment.

### 3.5. Influence of the BGs on ALP Activity

**Figure 6:**
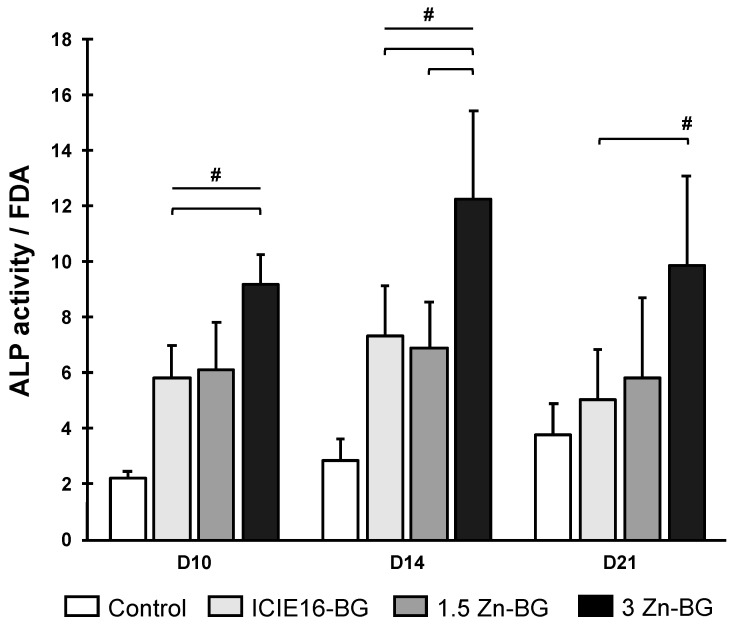
ALP activity. Fluorescent measurement of ALP activity was normalized on respective fluorescent measurement of FDA; both are shown in arbitrary units. Data are shown as means with standard deviation. Values labeled with # are significantly different compared to the control group. In case of multiple significant differences compared to the control group, the respective groups are labeled with a line and #. Brackets highlight significant differences between the two groups encompassed by the brackets.

The presence of the IDPs of all the BGs led to increased activity level of the ALP compared to that of the control group (Figure 6). At all times, the highest ALP activity level was measured in the group treated with 3Zn-BG. ALP activity increased with increasing Zn content of the BGs, suggesting a positive influence of Zn presence on ALP activity.

### 3.6. Gene Expression of Osteogenic Markers

**Figure 7:**
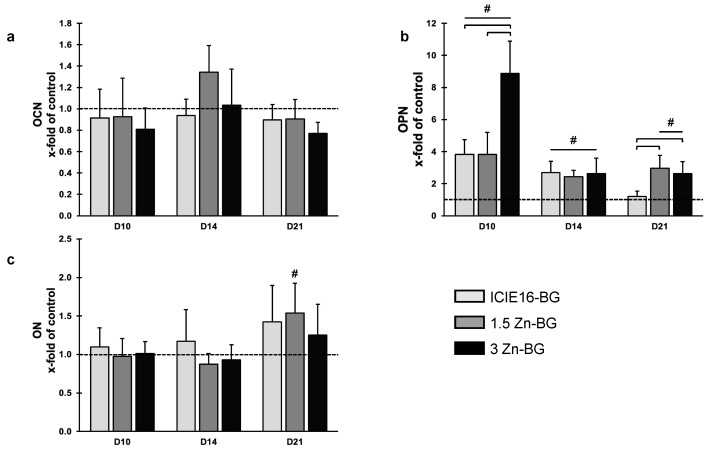
Gene expressions of (**a**) OCN, (**b**) OPN, and (**c**) ON are shown as x-fold of the control group (illustrated as dotted line). Data are shown as means with standard deviation. Values labeled with # are significantly different compared to the control group. In case of multiple significant differences compared to the control group, the respective groups are labeled with a line and #. Brackets highlight significant differences between the two groups encompassed by the brackets.

Whilst the OCN expression levels were only slightly regulated by the presence of the BG IDPs, the expression levels of OPN were significantly regulated by presence of the BGs on D10; the BGs increased the expression level significantly with the 3Zn group exceeding both the other groups (Figure 7a,b). On D14, no significant differences between the BG groups were detectable; however, all the groups exceeded the OPN expression levels compared to that of the control group significantly. On D21, the Zn-supplemented BGs induced OPN expression levels with significant differences to both the ICIE16-BG group and the control group. Regarding the expression levels of ON, no increase was noticed until D21 (Figure 7c). On D21, the presence of the IDPs increased the ON expression levels numerically when compared to that of the control group. A significant enhancement of expression was only detected in the 1.5Zn-BG group.

### 3.7. ECM Formation and Maturation in the Presence of Dilution Products

**Figure 8:**
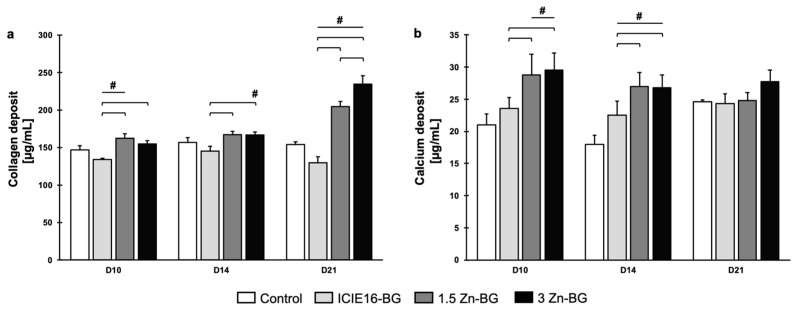
(**a**) Collagen deposition and (**b**) ECM calcification. Data are shown as means with standard deviation. Values labeled with # are significantly different compared to the control group. In case of multiple significant differences compared to the control group, respective groups are labeled with a line and #. Brackets highlight significant differences between the two groups encompassed by the brackets.

On D21, collagen deposition peaked especially when exposed to the 3Zn-BG IDPs, that outperformed every other group significantly (Figure 8a). The collagen deposition increased with an increasing Zn content in the BGs. The presence of ICIE16-BG IDPs decreased the level of collagen production compared to that of the control during the early stages of incubation, even to a significant extent. The addition of Zn strongly improved the ability of the BG to induce collagen deposition. Whilst the collagen production level was not increased by its presence, the ICIE16-BG had a no negative effects on the calcification of the ECM. Comparable to collagen production, the presence of Zn led to an increase in ECM calcification also in a concentration-dependent manner, with significant improvements compared to those of both the control group and the ICIE16-BG group on D10 and D14 (Figure 8b). On D21, ECM calcification leveled among all the groups with a low-level enhancement measured in the 3Zn-BG group.

## 4. Discussion

The biological properties of BGs can be shaped towards their anticipated field of application by the addition of ions with a known therapeutic activity [5]. The ICIE16-BG composition has already demonstrated improved cytocompatibility when compared to that of the 45S5-BG; however, its osteogenic properties are comparable [13]. Therefore, ICIE16-BG has been modified by the incorporation of ions that influence the osteogenic properties in a positive manner [12]. Recently, ZnO was added to the ICIE16-BG composition in exchange for CaO, resulting in two new BG compositions, namely 1.5Zn-BG and 3Zn-BG. In this study, the impact of Zn doping on the cytocompatibility and osteogenic properties of these new BGs were assessed in direct comparison to the unmodified ICIE16-BG.

Due to the known positive impact of Zn on the formation and mineralization of the osseous ECM [15,26,27], Zn constitutes a promising candidate ion to be added to the ICIE16-BG composition. In a recent study conducted by our group, the ICIE16-BG proved to increase the cellular osteogenic differentiation of BMSCs towards osteoblasts; however, its stimulating effects on the genes that encode for relevant proteins of the ECM were limited [13]. In order to assess the impact of the Zn-containing ICIE16-BG derivates on ECM formation and maturating using different assays, an indirect culture setting was applied, in which the BMSCs were grown in a preconditioned medium, but not in the physical presence of BGs [5]. This setting is established in biomaterials research and—transferred to the “in vivo” situation—allows one to study the impact of the IDPs on cells, which are not located in contact to the BGs, but in their surrounding environment without direct (physical) exposure [28]. Remarkably, the cell population that is exposed to the IDPs of BGs has shown to play a crucial role on the synthesis and maturation of the ECM [29]. Therefore, the indirect cultivation setting is suitable when assessing the effect of biomaterials on ECM formation and its maturation.

Due to their high bioreactivity, BGs exhibit a significant influence on their surrounding environment, especially in the early stages of contact with biological fluids [30]. These interactions, however, are necessary not only for the development of hydroxyapatite on the BG surface, but are also responsible for the suitability to tailor the biological properties of BGs by the addition (and release) of therapeutically active ions [5,6]. In this study, the presence of BG IDPs decreased the viability of the BMSCs compared to that of the untreated control group, whilst the addition of Zn to the ICIE16-BG composition did tendentially increase the cell viability especially during the later stages of cultivation. In the literature, different effects of the addition of Zn on the cytocompatibility of BGs have been described; in a previously published study conducted by our group, the addition of ZnO to mesoporous bioactive glass nanoparticles (MBGNs) in the system SiO_2_-CaO did change the BMSCs’ viability to a relevant extent [31]. On the other hand, there are also data showing a negative (decreasing) impact of Zn-containing BGs on the viability of both Saos-2 and MG-63 cells [32,33]. The Zn-dependent toxicity towards MG-63 cells was attributed to an environment of high-level oxidative stress resulting from the rapid release of Zn ions from the BGs [33]. Even the cells that are considered as being “alike”, such as the osteoblast-like cell lines Saos-2 and MG-63 that are frequently used to assess the biocompatibility of BGs, show significant differences in viability after exposure to BGs [34]. In this context, MG-63 cells appear to be more sensitive towards oxidative stress [35], while the BMSCs have potent compensatory mechanisms that make them less sensitive [36].

Within the used cell culture setting, the differentiation of BMSCs towards osteoblasts is characterized by an increase in ALP activity [37]. The ALP activity peaks usually after 10–14 days of cultivation [38]. The presence of the BGs’ IDPs led to an increased the ALP activity in comparison with that of the control; a peak was analyzed on D14, with a slight decrease until D21. Also, the presence of Zn significantly increased the ALP activity. Being a vital cofactor to ensure the enzymes’ function and activity, Zn, therefore, also affects osteogenic differentiation: for example, in 1986, Yamaguchi et al. described the positively correlated relationship between the presence of Zn and the activity of ALP [33,39]. Several other studies thereafter confirmed these findings also when Zn was used as a part of the biomaterials, such as BGs [26,39]. Whilst ALP is mainly used as a surrogate parameter for cellular osteogenic differentiation in cell culture settings, ALP plays also an important role in the mineralization of the ECM; via the hydrolysis of pyrophosphate, ALP provides free, inorganic phosphate, which subsequently mediates the mineralization of the ECM via the formation of hydroxyapatite [40,41]. Zn deficiency decreases ECM production and calcification, as shown by Alcantara et al. [42], outlining the significant role of Zn in osteogenic differentiation. In this study, ECM formation and maturation were enhanced by the presence of Zn, matching the observations of ALP activity evolution during the observation period and confirming the findings reported in the literature.

In contrast to the significant effects of Zn addition to the ICIE16-BG composition on ECM formation and maturation, the impact on the regulation of genes encoding for other protein members of the ECM was limited. For example, all the BGs had no relevant impact on OCN gene expression. These findings match the results reported by Wang et al., who did not find a relevant alteration of OCN gene expression when cultivating the BMSCs in an osteogenic differentiation medium [43]. Similar observations were made for ON gene expression. The BGs, however, did influence the gene expression levels of OPN that is more regulated in presence of Zn. OPN is committed in the regulation of bone mass, development, and maintenance [44].

Since BGs are an important part of daily clinical routines [45], this study identifies Zn substitution as beneficial for the osteogenic properties of the ICIE16-BG, especially when it comes to the BG’s influence on the formation and maturation of the ECM. However, there are some limitations that need to be included in interpretation of the data: It is known that the static, two-dimensional in vitro settings as used in this study do not represent the complex physiology of an organism; therefore, the transferability of the study results to the role of the Zn-based ICIE-16-BG in their anticipated field of applications is limited [46,47]. Also, in order to assess the influence of the BGs on ECM formation and maturation, an indirect cultivation setting was used, where the cells are grown in media enriched with the IDPs of the BGs, but not in their physical presence [28]. Therefore, the influence of the mechanisms on the BG surface that eventually lead to formation of a hydroxyapatite layer cannot be estimated based on the findings of this study; however, these mechanisms are relevant for the understanding of the biological properties of BGs, since the stimulation of certain biological effects depends on direct cell–BG contact [48]. Also, this study was designed as a stimulus–effect relationship approach in order to check if the addition of Zn to the ICIE16-BG composition leads to the desired effect on the ECM, which was the case. However, the mechanisms that lead to this effect have not been evaluated and should be the subject of further investigation.

Although a significant amount of further (experimental) data is still necessary to understand the biological properties of Zn-containing ICIE16-BGs in greater detail, Zn substitution and the ICIE16-BG composition should not only be considered for further experimental investigation, but can certainly be further investigated as potential candidates for “bench-to-bedside” transfer. However, it is necessary to evaluate the properties of the Zn-containing ICIE16-BG in greater detail not only in further in vitro experiments, but also using in vivo models, analyzing its influence on bone regeneration in more realistic settings [46,47].

## 5. Conclusions

In this study, the biological properties of the Zn-supplemented ICIE16-BG were examined evaluating cytocompatibility and the BGs’ impact on osteogenic differentiation, with a focus on the formation and maturation of the ECM. While Zn slightly improves the cytocompatibility of the ICIE16-BG composition, the presence of Zn significantly improves the osteogenic differentiation of BMSCs, and especially, their ability to build and calcify a primitive osseous ECM. The particularly positive influence of the Zn-substituted ICIE16-BG on the formation and maturation of the ECM qualifies these BGs compositions for future research. The upcoming studies should focus on the clarification of the cellular mechanisms behind the observed effects, in particular, in terms of Zn concentration, and should analyze the impact of the Zn containing BGs on bone regeneration using appropriate in vivo models.

## Figures and Tables

**Table 1:** Chemical composition of bioactive glasses in mol%.

Glass	SiO_2_	CaO	P_2_O_5_	Na_2_O	K_2_O	ZnO
ICIE16-BG	49.46	36.27	1.07	6.60	6.60	-
1.5Zn-BG	49.46	34.77	1.07	6.60	6.60	1.50
3Zn-BG	49.46	33.27	1.07	6.60	6.60	3.00

**Table 2:** Primers used for qPCR. Shown sequences were used to detect the activity of genes that encode for the proteins Osteopontin (OPN), Osteocalcin (OCN), Osteonectin (ON), and Tryptophan 5-Monooxygenase Activation Protein Zeta (YWHAZ).

Gene	Forward Strand (5′ → 3′)	Reverse Strand (5′ → 3′)
YWHAZ	TGC TTG CAT CCC ACA GAC TA	AGG CAG ACA ATG ACA GAC CA
OPN	GCT AAA CCC TGA CCC ATC TC	ATA ACT GTC CTT CCC ACG GC
OCN	ACC GAG ACA CCA TGA GAG CC	GCT TGG ACA CAA AGG CTG CAC
ON	TTC CCT GTA CAC TGG CAG TTC	AAT GCT CCA TGG GGA TGA

## Data Availability

All the relevant data are shown within the article.
